# Supplementary material for: B7-CD28 co-stimulation modulates central tolerance via thymic clonal deletion and Treg generation through distinct mechanisms
Source: Nat Commun. 2020 Dec 8;11:6264. doi: 10.1038/s41467-020-20070-x (PMC7722925; doi:10.1038/s41467-020-20070-x)
Supplement: Supplementary file 1 — Supplementary Information [file 41467_2020_20070_MOESM1_ESM.pdf]

SUPPLEMENTARY INFORMATION

B7-CD28 co-stimulation modulates central tolerance via thymic clonal deletion and Treg generation through distinct mechanisms

Masashi Watanabe[1], Ying Lu[1], Michael Breen[1] and Richard J. Hodes[1]*

1 Experimental Immunology Branch, National Cancer Institute, Bethesda, MD 20852, USA

* Corresponding author: Richard J. Hodes (hodesr@31.nia.nih.gov).

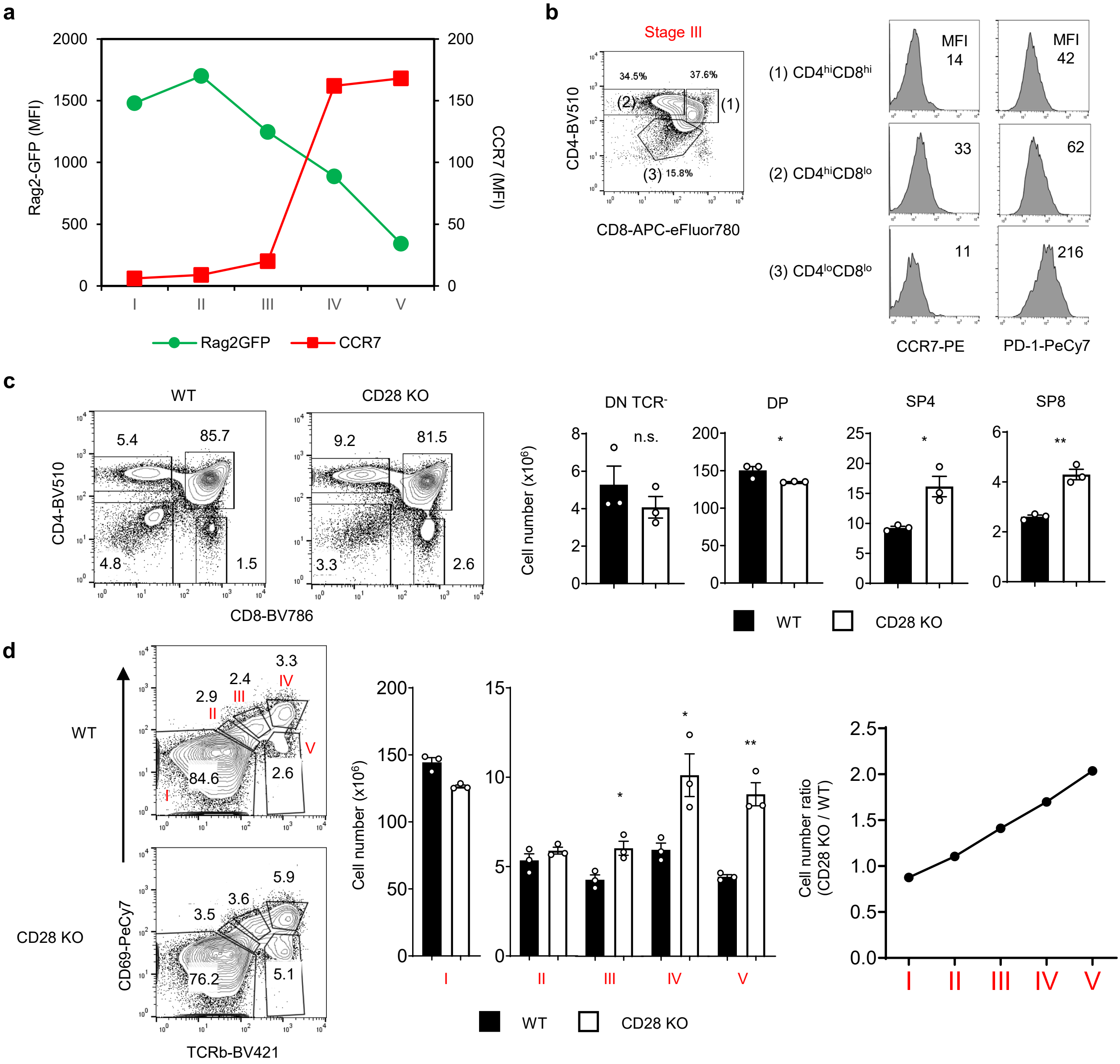

Supplementary Fig. 1 Thymocyte subsets phenotype and clonal deletion of CD28 KO mice. (a) Rag2-GFP and CCR7 expression (MFI) in each stage of thymic developmental. (b) CCR7 and PD-1 expression (MFI) of Stage III subsets. Data are representative result of three independent experiments. (c) Cell number and frequency of thymocyte subsets in WT and CD28 KO mice. WT n =3, CD28 KO n = 3. DP p = 0.0485, SP4 p = 0.0159, SP8 p = 0.0015. Data are representative results of at least three independent experiments. (d) Thymocyte developmental stages defined by TCR vs. CD69 expression (left). Thymocyte number in each developmental stage (middle). Stage III p = 0.0209, Stage IV p = 0.290, Stage V p = 0.0022. Ratio of relative thymocyte number in CD28 KO to WT mice in each stage (right). Each group n = 3. Data are representative results of two independent experiments. Data are mean ± SEM with dots representing individual values of biologically independent animals. Statistical differences between groups were calculated using unpaired, two tailed Student's t-test. * $p < 0.05$, ** $p < 0.01$. n.s.; not significant ($p > 0.05$). Source data are provided as a Source Data file.

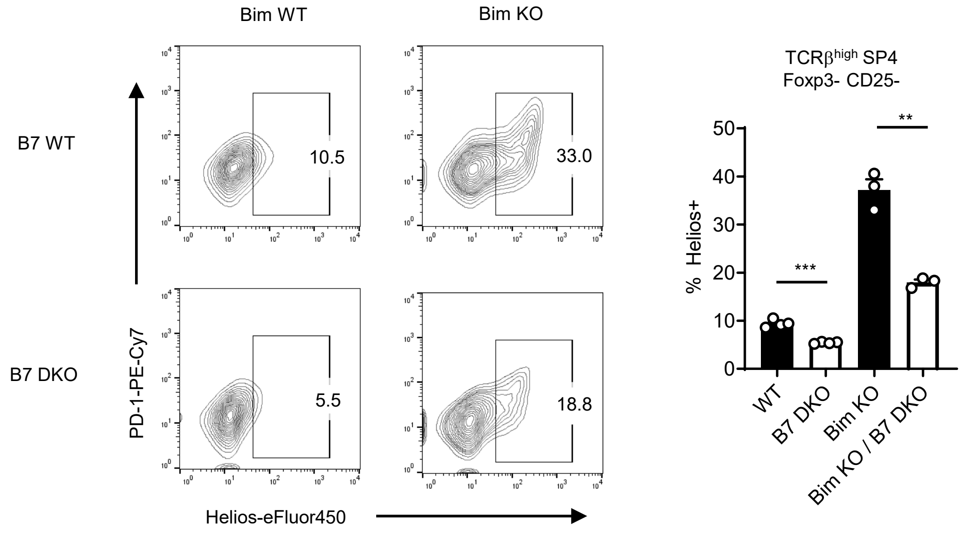

Supplementary Fig. 2 Clonal deletion of SP4 in Bim and B7 DKO thymus. Decreased frequency of clonally deleting cells (Helios^{+}) in SP4 Tconv (CD25^{-}/Foxp3^{nega}) cells in Bim (*Bcl2l11*) KO / B7 DKO compared to Bim KO. WT n = 4, B7 DKO n = 4, Bim KO n = 3, Bim KO / B7 DKO n = 3. WT and B7 DKO p < 0.0001, Bim KO and Bim / B7 TKO p = 0.0011. Data are pooled results of two independent experiments. Data are mean ± SEM with dots representing individual values of biologically independent animals. Statistical differences between groups were calculated using unpaired, two tailed Student's t-test. ** p < 0.01, *** p < 0.001. Source data are provided as a Source Data file.

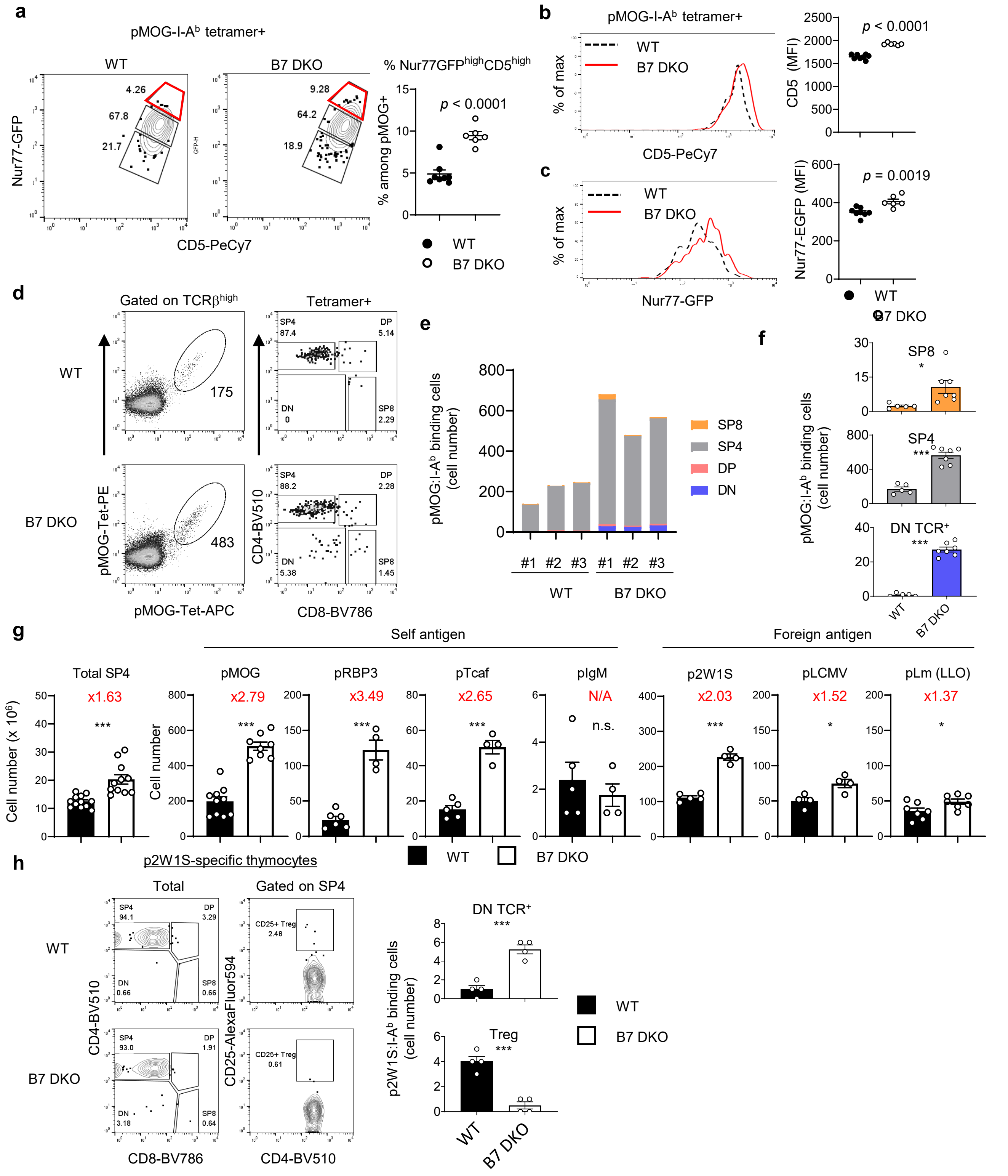

Supplementary Fig. 3 Clonal diversion of MOG-specific thymocytes. (a, b, c) pMOG-specific SP4 cells were analyzed for Nur77-GFP and CD5 expression. WT n = 8, B7 DKO n = 6. Data are representative results of two independent experiments. (d, e) Thymocyte subset composition among pMOG-binding cells in WT and B7 DKO mice. Data are representative results of at least three independent experiments. FACS plots are analysis for tetramer enriched cells gated on Thy1.2^{+} Lineages^{-} and TCR^{high} cells, as described in the Methods. (f) Quantitative analysis of each subset depicted in *(e)*. Data were pooled from two independent experiments. SP8 $p = 0.0345$, SP4 $p < 0.0001$, DN TCR^{+} $p < 0.0001$. WT n = 5, B7 DKO n = 7. (g) Summary of antigen-specific CD4SP Tconv cell number for multiple self and nominally foreign antigens. Red-highlighted number is fold change of average cell number (B7 DKO / WT). Total SP $p = 0.0002$, pMOG $p < 0.0001$, pRBP3 $p < 0.0001$, pTcaf $p < 0.0001$, p2W1S $p < 0.0001$, pLCMV $p = 0.0168$, pLm (LLO) $p = 0.0365$. Total SP: WT n = 12, B7 DKO n = 11; pMOG: WT n = 10, B7 DKO n = 8; pRBP3: WT n = 6, B7 DKO n = 4; pTcaf: WT n = 5, B7 DKO n = 4; pIgM: WT n = 5, B7 DKO n = 4; p2W1S: WT n = 5, B7 DKO n = 4; pLCMV: WT n = 4, B7 DKO n = 4; pLm (LLO): WT n = 7, B7 DKO n = 7. Data were pooled from 2-5 independent experiments. (h) p2W1S-specific Treg and DN TCR^{+} cells in WT and B7 DKO thymus. WT n = 4, B7 DKO n = 4. DN TCR^{+} $p = 0.0005$, Treg $p = 0.0004$. Data are representative result of at least 3 independent experiments. Data are mean ± SEM with dots representing individual values of biologically independent animals. Statistical differences between groups were calculated using unpaired, two tailed Student's t-test. * $p < 0.05$, ** $p < 0.01$, *** $p < 0.001$. n.s.; not significant ($p > 0.05$). Source data are provided as a Source Data file.

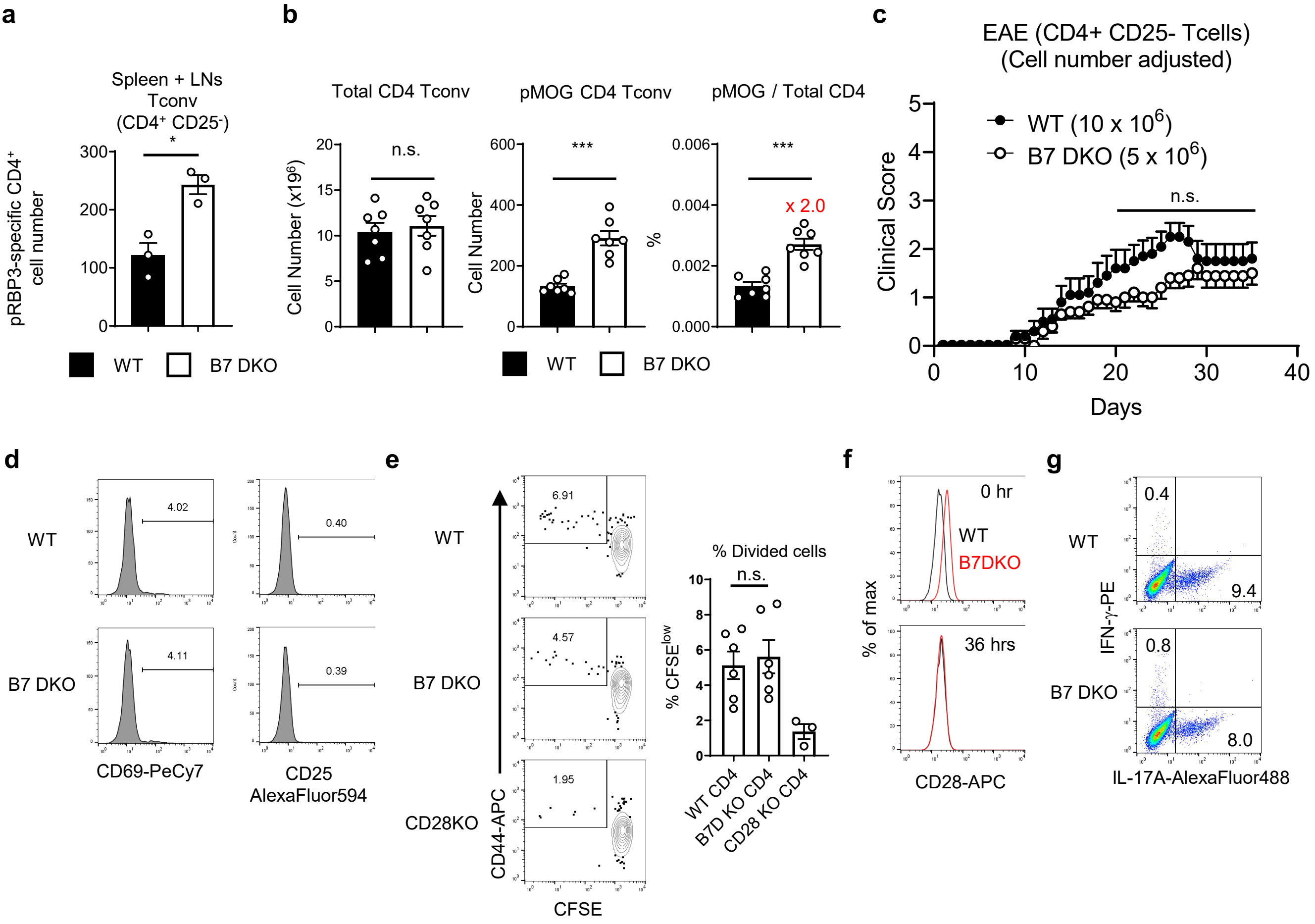

Supplementary Fig. 4 Peripheral TRA-specific and total CD4⁺ T cells. **a** Number of pRBP3-specific CD4⁺ T cells in pooled spleen and LNs. Each group n = 3. *p* = 0.0106. Data are representative results of two independent experiments. **b** Splenic CD4 Tconv cell number, pMOG-specific CD4⁺ T cell number and frequency. Total CD4+ Tconv cell number was not different between WT and B7 DKO. Average of pMOG Tconv frequency in total Tconv was two-fold increased in B7 DKO compared to WT. Data were pooled from three independent experiments. pMOG-CD4-Tconv $p < 0.0001$, pMOG/Total $p < 0.0001$. WT n = 7, B7 DKO n = 7. (**a, b**) Data are mean ± SEM with dots representing individual values of biologically independent animals. Statistical differences between groups were calculated using unpaired, two tailed Student's t-test. **c** EAE was induced with adjusted transferring T cell number to contain roughly equal number of pMOG-specific CD4⁺ T conv cells. Both groups n = 10 biologically independent animals. Medians of total clinical score during day 20 – 35 were compared by two-tailed non-parametric Mann-Whitney test. Data are representative result of two independent experiments. **d** Naïve CD4 Tconv cells ($CD44^{low}$, $CD25^{-}$) enriched from WT and B7 DKO mice (CD45.2) were transferred to CD45.1 hosts and CD69 and CD25 expression levels were analyzed after 36 hours. Data are representative result of two independent experiments. **e** Naïve CD4 Tconv cells enriched from WT and B7 DKO mice were CFSE stained and transferred to CD45.1 hosts. CFSE dilution and CD44 expression were analyzed after one week. Data were pooled from two independent experiments. WT n = 6, B7 DKO n = 6, CD28 KO = 3. Data are mean ± SEM with dots representing individual values of biologically independent animals. Statistical differences between groups were calculated using unpaired, two tailed Student's t-test. **f** CD28 expression levels before and after adoptive transfer. Naïve CD4 Tconv cells enriched from WT and B7 DKO mice were transferred to CD45.1 hosts and CD28 expression level was analyzed after 36 hours. Data are representative result of two independent experiments. **g** In vitro Th17 differentiation. Naïve CD4 Tconv cells enriched from WT and B7 DKO mice were stimulated with anti-CD3 plus anti-CD28 in the presence of IL-6, TGFβ , anti-IFNγ and anti-IL-4 for 5 days. The cells were re-stimulated with PMA plus Ionomycin for 4 hours in the presence of monensin and intracellular cytokine staining was performed. Data were representative result of three independent experiments. * $p < 0.05$, *** $p < 0.001$. n.s.; not significant ($p > 0.05$). Source data are provided as a Source Data file.

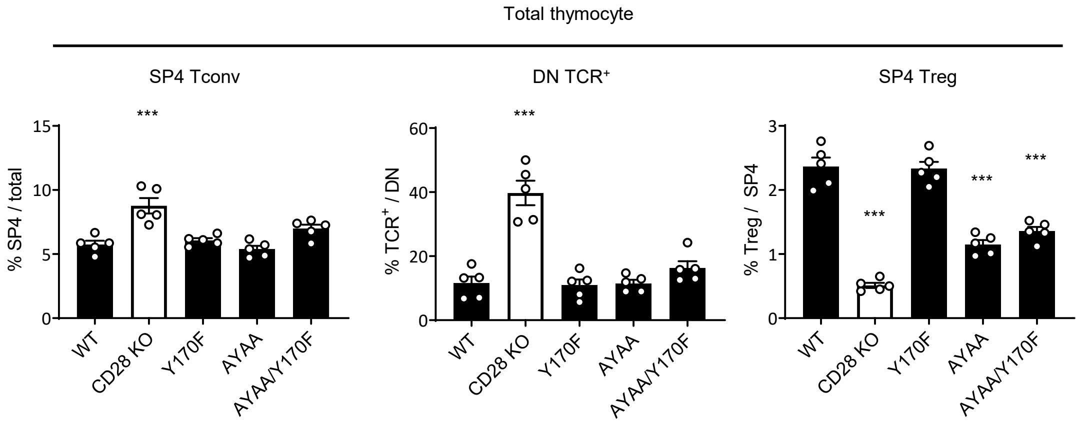

Supplementary Fig. 5 CD28 signal domain requirement for thymocyte subsets development at population level. Frequency of total SP4 Tconv (Foxp3$^-$, CD25$^-$) cells among total thymocytes (left), frequency of TCRβ$^+$ among DN (middle) and frequency of total SP4 Treg (Foxp3$^+$, CD25$^+$) cells among SP4 (right) in each CD28 mutant strain. Each group n = 5. Data shown are combined results of three independent experiments. SP4 Tconv (CD28 KO p < 0.0001), DN TCR$^+$ (CD28 KO p = 0.0008), SP4 Treg (CD28KO $p < 0.001$, AYAA $p < 0.0001$, AYAA/Y170F $p < 0.0001$). Data are mean ± SEM with dots representing individual values of biologically independent animals. Statistical differences between groups was performed to WT with One-way ANOVA followed by Dunnett's multiple comparison. *** $p < 0.001$. Source data are provided as a Source Data file.

a

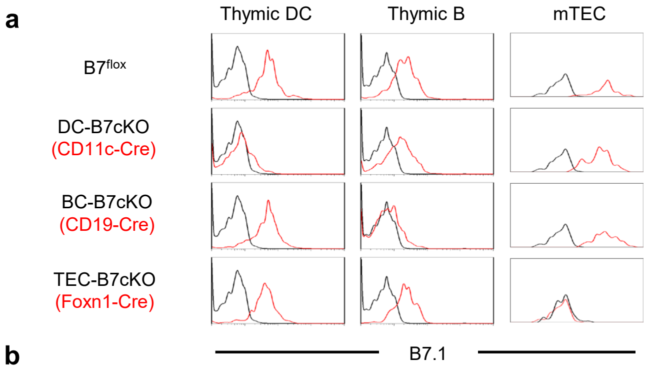

b

Strain name (all B7.1/B7.2 DKO background)	$B7.1^{flox}$ BAC Tg	Cre-Tg	B7 expression on thymic APCs	Label	
$B7^{flox}$	+	-	DC, B cell, TEC	$B7^{flox}$	
B7 DKO	-	-	~~DC~~, B ~~cell~~, ~~TEC~~	B7 DKO	
DC/BC/TEC-B7cKO	+	CD11c-Cre CD19-Cre Foxn1-Cre	~~DC~~, B ~~cell~~, ~~TEC~~	DC/BC/TEC-	
DC-B7cKO	+	CD11c-Cre	~~DC~~, B cell, TEC	DC-	One APC lacks B7 (Necessity)
BC-B7cKO	+	CD19-Cre	DC, B ~~cell~~, TEC	BC-	
TEC-B7cKO	+	Foxn1-Cre	DC, B cell, ~~TEC~~	TEC-	
DC/TEC-B7cKO	+	CD11c-Cre Foxn1-Cre	~~DC~~, B cell, ~~TEC~~	BC+	One APC expresses B7 (Sufficiency)
DC/BC-B7cKO	+	CD11c-Cre CD19-Cre	~~DC~~, B ~~cell~~, TEC	TEC+	
BC/TEC-B7cKO	+	CD19-Cre Foxn1-Cre	DC, B ~~cell~~, ~~TEC~~	DC+	

c

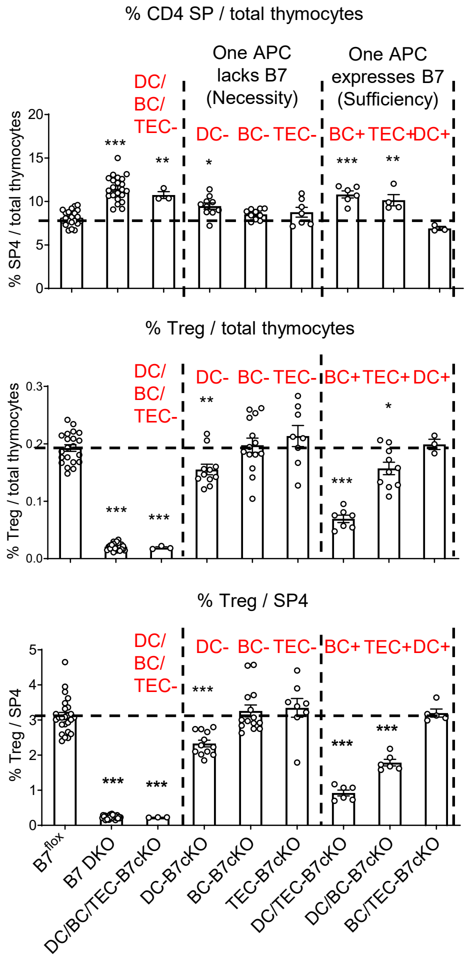

f

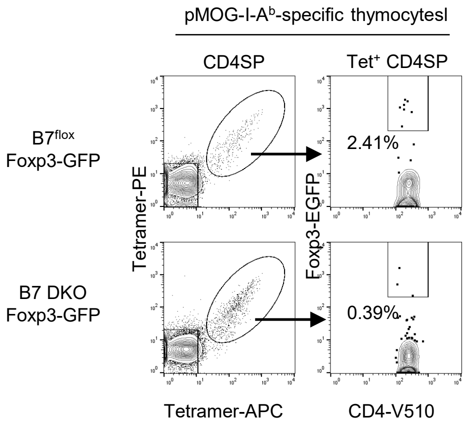

g

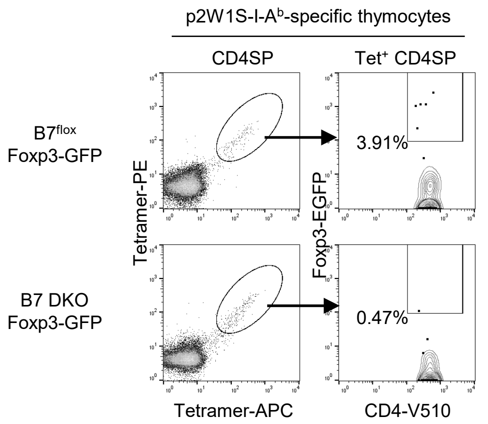

Supplemental Fig. 6 Cellularity of SP4 Tconv and Treg cells in B7 conditional KO strains. **a** B7-deletion specificity in each Cre-Tg strain. **b** B7 cKO strain panel showing cell type(s) from which B7 is deleted. **c** Frequency of SP4 cells in each B7 cKO strain thymus. $p < 0.0001$ (B7 DKO, BC+), $p = 0.0028$ (DC/BC/TEC-), 0.0204 (DC-), 0.0124 (TEC+). n = 21 ($B7^{flox}$), 22 (B7 DKO), 3 (DC/BC/TEC-), 10 (DC-), 10 (BC-), 7 (TEC-), 6 (BC+), 4 (TEC+), 3 (DC+). **d** Frequency of Treg among total thymocytes in each B7 cKO strain. $p < 0.0001$ (B7 DKO, BC+), $p = 0.0001$ (DC/BC/TEC-), 0.0058 (DC-), 0.0108 (TEC+). n = 21 ($B7^{flox}$), 26 (B7 DKO), 3 (DC/BC/TEC-), 11 (DC-), 14 (BC-), 8 (TEC-), 7 (BC+), 10 (TEC+), 3 (DC+). **e** Frequency of Treg among SP4 in each B7 cKO strain thymus. $p < 0.0001$ (B7 DKO, DC/BC/TEC-, DC-, BC+, TEC+). n = 25 ($B7^{flox}$), 27 (B7 DKO), 3 (DC/BC/TEC-), 12 (DC-), 14 (BC-), 8 (TEC-), 6 (BC+), 6 (TEC+), 5 (DC+). (**c**-**e**) Data are combined results of at least 3 independent experiment. Data are mean ± SEM with dots representing individual values of biologically independent animals. Statistical analysis was performed for comparison to $B7^{flox}$ mice by one-way ANOVA followed by Dunnett's multiple comparison. **f** pMOG-specific SP4 Tconv ($Foxp3GFP^-$) and Treg ($Foxp3GFP^+$) cells in $B7^{flox}$ and B7 DKO mice. Data are representative results of at least 3 independent experiments. **g** p2W1S-specific SP4 Tconv ($Foxp3GFP^-$) and Treg ($Foxp3GFP^+$) cells in $B7^{flox}$ and B7 DKO mice. Data are representative results of at least 3 independent experiments. * $p < 0.05$, ** $p < 0.01$, *** $p < 0.001$. n.s.; not significant ($p > 0.05$). Source data are provided as a Source Data file.

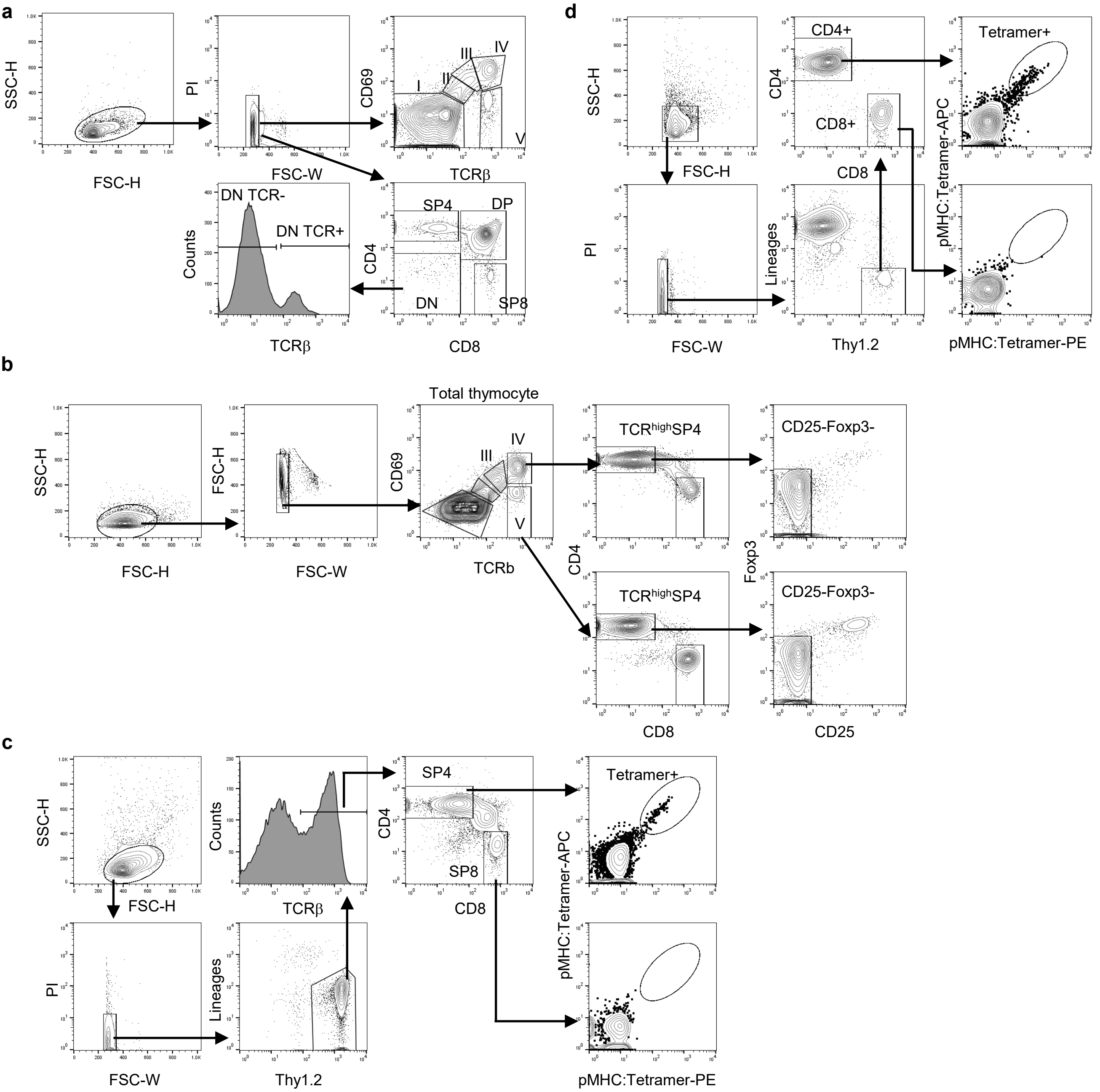

Supplemental Fig. 7 **Gating strategy for flow cytometry analysis.** **a** Gating strategy used to identify thymocytes subsets in Fig. 1, Fig. 2e and 2f. **b** Gating strategy used to identify thymocyte subsets for intracellular staining in Fig. 2a, 2b, 3c and 2d. **c** Gating strategy used to identify tetramer binding thymocytes after MACS enrichment in Fig. 3, Fig. 5 and Fig. 6. **d** Gating strategy used to identify tetramer binding peripheral T cells after MACS enrichment in Fig. 4. Lineage markers were B220, CD11c, CD11b, Gr1, NK1.1. Tetramer positive gate was defined by using SP8 ($CD4^-$, $CD8^+$) thymocytes or $CD8^+$ peripheral T cells as negative staining control.
