# Supplementary material for: B7-CD28 co-stimulation modulates central tolerance via thymic clonal deletion and Treg generation through distinct mechanisms
Source: Nat Commun. 2020 Dec 8;11:6264. doi: 10.1038/s41467-020-20070-x (PMC7722925; doi:10.1038/s41467-020-20070-x)
Supplement: Supplementary file 3 — Reporting Summary [file 41467_2020_20070_MOESM3_ESM.pdf]

nature research

Corresponding author(s): Richard J. Hodes
Last updated by author(s): Nov 2, 2020

Reporting Summary

Nature Research wishes to improve the reproducibility of the work that we publish. This form provides structure for consistency and transparency in reporting. For further information on Nature Research policies, see our Editorial Policies and the Editorial Policy Checklist.

Statistics

For all statistical analyses, confirm that the following items are present in the figure legend, table legend, main text, or Methods section.

n/a	Confirmed	
☐	☒	The exact sample size (*n*) for each experimental group/condition, given as a discrete number and unit of measurement
☐	☒	A statement on whether measurements were taken from distinct samples or whether the same sample was measured repeatedly
☐	☒	The statistical test(s) used AND whether they are one- or two-sided<br>*Only common tests should be described solely by name; describe more complex techniques in the Methods section.*
☒	☐	A description of all covariates tested
☐	☒	A description of any assumptions or corrections, such as tests of normality and adjustment for multiple comparisons
☐	☒	A full description of the statistical parameters including central tendency (e.g. means) or other basic estimates (e.g. regression coefficient) AND variation (e.g. standard deviation) or associated estimates of uncertainty (e.g. confidence intervals)
☐	☒	For null hypothesis testing, the test statistic (e.g. *F*, *t*, *r*) with confidence intervals, effect sizes, degrees of freedom and *P* value noted<br>*Give P values as exact values whenever suitable.*
☒	☐	For Bayesian analysis, information on the choice of priors and Markov chain Monte Carlo settings
☒	☐	For hierarchical and complex designs, identification of the appropriate level for tests and full reporting of outcomes
☒	☐	Estimates of effect sizes (e.g. Cohen's *d*, Pearson's *r*), indicating how they were calculated

Our web collection on statistics for biologists contains articles on many of the points above.

Software and code

Policy information about availability of computer code

Data collection	BD Bioscience FACS DIVA
Data analysis	TreeStar FlowJo (ver.10), GraphPad Prism (Ver.7)

For manuscripts utilizing custom algorithms or software that are central to the research but not yet described in published literature, software must be made available to editors and reviewers. We strongly encourage code deposition in a community repository (e.g. GitHub). See the Nature Research guidelines for submitting code & software for further information.

Data

Policy information about availability of data

All manuscripts must include a data availability statement. This statement should provide the following information, where applicable:

- Accession codes, unique identifiers, or web links for publicly available datasets
- A list of figures that have associated raw data
- A description of any restrictions on data availability

The data supporting the key findings of this study are available within the article and its Supplementary Information files or from the corresponding author upon reasonable request. Source data are provided as a Source Data file with this paper.

Field-specific reporting

Life sciences study design

All studies must disclose on these points even when the disclosure is negative.

Sample size	No statistical method was used to predetermine sample size. Sample size was determined to be adequate based on previous experience with similar experiments and on the magnitude and consistency of measurable differences between groups in order to reproducibly detect specific effects. Sample sizes varies as indicated in figure legends.
Data exclusions	No data were excluded from analyses.
Replication	At least three independent biologic replicates were carried out for each experiment. All attempts to replicate data were successful.
Randomization	Mice were sex and age matched for tested and control samples.
Blinding	Investigators were not blinded to mouse genotypes for planning of experiments and to ensure that appropriate sample size was achieved by sacrificing the minimum number of mice. However, clinical score of EAE experiments were scored by an investigator in a mice genotype/group-blinded manner to prevent bias in research.

Reporting for specific materials, systems and methods

We require information from authors about some types of materials, experimental systems and methods used in many studies. Here, indicate whether each material, system or method listed is relevant to your study. If you are not sure if a list item applies to your research, read the appropriate section before selecting a response.

Materials & experimental systems

n/a	Involved in the study
☐	☒ Antibodies
☒	☐ Eukaryotic cell lines
☒	☐ Palaeontology and archaeology
☐	☒ Animals and other organisms
☒	☐ Human research participants
☒	☐ Clinical data
☒	☐ Dual use research of concern

Methods

n/a	Involved in the study
☒	☐ ChIP-seq
☐	☒ Flow cytometry
☒	☐ MRI-based neuroimaging

Antibodies

Antibodies used

Antibody, Clone, Manufacture, Catalog #, Dilution.
Anti-CD4-BV510, RM4-5, BD Bioscience, 563106, 1:200
Atni-CD8-BV786, 53-6-7, BD Bioscience, 563332, 1:200
Atni-CD8-APC-eFluor780, 53-6-7, eBioscience 47-0081-82, 1:200
Anti-CD5-APC 53-7-3, eBioscience, 17-0051-82 1:400,
Anti-CCR7-PE, 4B12, BD Bioscience, 560682, 1:200
Anti-CD11c-APC-eFluor780, N418, eBioscience, 47-0114-82, 1:200
Atni-CD11b-APC-eFluor780, M1/70, eBioscience, 47-0112-82, 1:200
Atni-CD25-AlexaFluor488, PC61, eBioscience, 53-0251-82 1:200
Atni-CD44-AlexaFluor488, IM7, BioLegend, 103015 1:400
Atni-CD69-PE, H1.2F3 BioLegend, 104507, 1:200
Atni-CD73-Biotin, TY/11.8, BioLegend, 127203, 1:200
Atni-FR4-PeCy7, 12A5, BioLegend, 125012, 1:200
Atni-Gr1-APC-eFluor780, RB6-8C5, eBioscience, 47-5931-82, 1:200
Atni-NK1.1-APC-eFluor780, PK136, eBioscience, 47-5941-82, 1:200
Atni-B220-APC-eFluor780, RA3-6B2, eBioscience, 47-0452-82, 1:200
Atni-B7.1-APC, 16-10A1, eBioscience, 17-0801-82, 1:100
Atni-PD-1-PeCy7, 29F.1A12, BioLegend, 135215, 1:200
Atni-TCRb-APC, H57-597, eBioscience, 17-5961-82, 1:200
Atni-TCRb-BV421, H57-597, BioLegend, 109229, 1:200
Atni-Thy1.2-AlexaFluor700, 30-H12, BioLegend, 105319, 1:400
Atni-Foxp3-AlexaFluor488, FJK-16s, eBioscience, 12-5773-82, 1:50
Atni-Foxp3-AlexaFluor700, FJK-16s, eBioscience, 56-5773-82, 1:50
Atni-Foxp3-eFluor450, FJK-16s, eBioscience 48-5773-82, 1:50
Atni-Helios-eFluor450, 22F6, eBioscience, 48-9883-42, 1:50
Atni-Active Caspase3-PE, D3E9, Cell Signaling, 12768S, 1:50

Validation	All antibodies used are commercially available, and were validated with mouse cells for flow cytometry application by the manufacturers. Validation statement for each antibody is found on manufacture's websites.<br>BD Bioscience (https://www.bdbiosciences.com/)<br>ThermoFisher/eBiosience (https://www.thermofisher.com/us/en/home/life-science/antibodies/ebioscience.html)<br>BioLegends (https://www.biolegend.com/)<br>Cell Signaling (https://www.cellsignal.com/)<br><br>Upon receipt, antibodies were validated in the laboratory for flow cytometry application by using mouse thymocyte and splenocyte populations known to be positive and negative staining and using isotype control antibodies.

Animals and other organisms

Policy information about studies involving animals; ARRIVE guidelines recommended for reporting animal research

Laboratory animals	C57BL/6 (B6), B7.1/B7.2 double KO (B7 DKO), CD28 KO, Aire KO, TCRalpha KO, IgM KO, Bim KO, CD11c-Cre-Tg, CD19-Cre knockin, Nur77-GFP Tg, Foxn1-Cre-Tg, B7.1flox BAC Tg / B7 DKO, Foxp3-GFP knock-in, CD28-Y170F, CD28-AYAA and CD28-Y170F/AYAA knock-in mice, CD28-WT and CD28-TL Tg, Bcl2-Tg 75, Rag2-GFP-Tg mice, female and male, age between 5 - 8 weeks. Mice were bred and maintained in our specific pathogen free (SPF) animal facility, at ambient temperature 22 ± 2 °C, humidity 50 ± 20%, and a dark/light cycle of 12 h daily, in accordance with US National Institutes of Health guidelines.
Wild animals	This study did not use wild animals.
Field-collected samples	This study did not use field-collected samples.
Ethics oversight	All animal experiments were approved by the NCI and Animal Care and Use Committees and carried out according to the National Institutes of Health Guide for Care and Use of Laboratory Animals.

Note that full information on the approval of the study protocol must also be provided in the manuscript.

Flow Cytometry

Plots

Confirm that:

- [x] The axis labels state the marker and fluorochrome used (e.g. CD4-FITC).
- [x] The axis scales are clearly visible. Include numbers along axes only for bottom left plot of group (a 'group' is an analysis of identical markers).
- [x] All plots are contour plots with outliers or pseudocolor plots.
- [x] A numerical value for number of cells or percentage (with statistics) is provided.

Methodology

Sample preparation	Cells from mouse thymus, spleen and lymph node were isolated by manual disruption into buffer as described in the Method.
Instrument	BD Bioscience FACS Fortessa, FACS LSR II, FACS Aria III.
Software	BD Bioscience FACS DIVA for data collection, TreeStar FlowJo for data analysis.
Cell population abundance	This study did not include FACS sort experiment.
Gating strategy	Cells were initially gated by size based on FSC(H)/SSC, followed by FSC(W) to exclude cell doublets. Live cells were then gated based on viability dye before analysis for cellular markers.

- [x] Tick this box to confirm that a figure exemplifying the gating strategy is provided in the Supplementary Information.
